# Astronomical pacing of the global silica cycle recorded in Mesozoic bedded cherts

**DOI:** 10.1038/ncomms15532

**Published:** 2017-06-07

**Authors:** Masayuki Ikeda, Ryuji Tada, Kazumi Ozaki

**Affiliations:** 1 Department of Geosciences, Graduate School of Science, Shizuoka University, 836 Ooya, Suruga-ku, Shizuoka 790-8577, Japan; 2 Lamont Doherty Earth Observatory, Columbia University, Columbia, New York 10964, USA; 3 Department of Earth and Planetary Science, Graduate School of Science, University of Tokyo, Tokyo 113-8654, Japan; 4 School of Earth and Atmospheric Sciences, Georgia Institute of Technology, Atlanta, Georgia 30332, USA; 5 NASA Postdoctoral Program, Universities Space Research Association, Columbia, Maryland 21046, USA

## Abstract

The global silica cycle is an important component of the long-term climate system, yet its controlling factors are largely uncertain due to poorly constrained proxy records. Here we present a ∼70 Myr-long record of early Mesozoic biogenic silica (BSi) flux from radiolarian chert in Japan. Average low-mid-latitude BSi burial flux in the superocean Panthalassa is ∼90% of that of the modern global ocean and relative amplitude varied by ∼20–50% over the 100 kyr to 30 Myr orbital cycles during the early Mesozoic. We hypothesize that BSi in chert was a major sink for oceanic dissolved silica (DSi), with fluctuations proportional to DSi input from chemical weathering on timescales longer than the residence time of DSi (<∼100 Kyr). Chemical weathering rates estimated by the GEOCARBSULFvolc model support these hypotheses, excluding the volcanism-driven oceanic anoxic events of the Early-Middle Triassic and Toarcian that exceed model limits. We propose that the Mega monsoon of the supercontinent Pangea nonlinearly amplified the orbitally paced chemical weathering that drove BSi burial during the early Mesozoic greenhouse world.

## Abstract (web summary)

While the global silica cycle is known to play an important role in long-term climate change, the driving factors remain unknown. Here, the authors present a ∼70 million year long record of early Mesozoic biogenic silica and propose orbitally-paced chemical weathering as a primary driver.

The global silica cycle regulates long-term (>10^5^ yr) changes in Earth's climate through the negative feedback mechanism that operates between atmospheric CO_2_, climate and the rate of silicate weathering followed by carbonate and biogenic silica (BSi) deposition^1^. Changes in Si and C cycle dynamics are linked to global climate changes throughout Earth history, a relationship, which in turn, allows numerical models to reconstruct past atmospheric *p*CO_2_ (ref. 2). Understanding the nature of the global silica cycle is therefore crucial to elucidate the functionality of Earth's surface system to changes in external forcings. Orbital forcing is one of the main pace makers of biogeochemical cycles such as silicate weathering, not only through glaciation, but also through monsoonal precipitation mainly during the Cenozoic^3^. However, the impact of orbital forcing on biogeochemical cycles is difficult to quantify and arguably poorly understood due to large uncertainty in the proxy records, especially on longer timescales^3^.

The rhythmic deposition of early Mesozoic radiolarian bedded chert provides a rare record of pelagic deep-sea siliceous sediments before the Cretaceous^4^ that allows exploration of the impact of orbital forcing on the global biogeochemical silica cycle^5^. Bedded chert is formed as a result of tens of thousands- to multimillion-year scale changes in BSi burial flux, which are manifested as chert bed thickness for each precession cycle^5^; however, the underlying mechanisms for the cyclic changes in the BSi burial flux remain unknown.

The mechanisms that formed bedded chert need to be considered in the context of the global silica cycle. Hori *et al*.^6^ and De Wever *et al*.^7^ proposed that the orbitally paced variations in upwelling were linked to changes in productivity in the equatorial ocean, consistent with climate model simulation^8^. Although spatio-temporal variations in upwelling intensity would modulate the spatial distribution of BSi burial, the BSi burial rate on the global scale should have reflected variations in the input of dissolved silica (DSi) to the ocean on timescales longer than the residence time of DSi, which is *ca.* 10 kyr for the modern^9^ and <100 kyr for the Phanerozoic^10^. In the modern ocean, BSi of diatoms and siliceous sponges is the major sink for DSi, whereas BSi of radiolarians is unknown but likely significantly less than that of diatoms^10^. As the ecological rise of diatoms post dates the Cretaceous, radiolarians would have been a relatively larger sink of BSi before the Cretaceous^11^. It is therefore reasonable to suppose that bedded chert was a major repository for DSi in the early Mesozoic ocean before diatom biomass became important. If this was the case, then BSi burial fluxes in the form of bedded chert would have been controlled by the input flux of DSi to the ocean, mainly through global chemical weathering on land^9^, which could have been modulated by the orbital-scale summer monsoon dynamics^12^.

Here we reconstruct temporal variations in the BSi burial flux of the low- to mid-latitude pelagic Panthalassa superocean (hereafter, Panthalassa) during the early Mesozoic, using an ∼70 Myr record of cyclostratigraphically age-controlled bedded chert in the Inuyama area of central Japan^13^ (Fig. 1). To explore the possibility that the radiolarian BSi in bedded chert was a major sink for DSi, we evaluate the global BSi burial flux preserved in the Mesozoic bedded cherts and compare it with that in the modern global ocean. We further compare the temporal variations in BSi burial flux in Inuyama bedded cherts with global silicate weathering rates calculated from the latest GEOCARBSULFvolc model^14^,^15^ with some modifications (see Methods) and Sr isotopic trends in seawater as a proxy for global chemical weathering during the early Mesozoic^16^.

## Results

### BSi burial flux in the pelagic Panthalassa superocean

**Figure 1:**
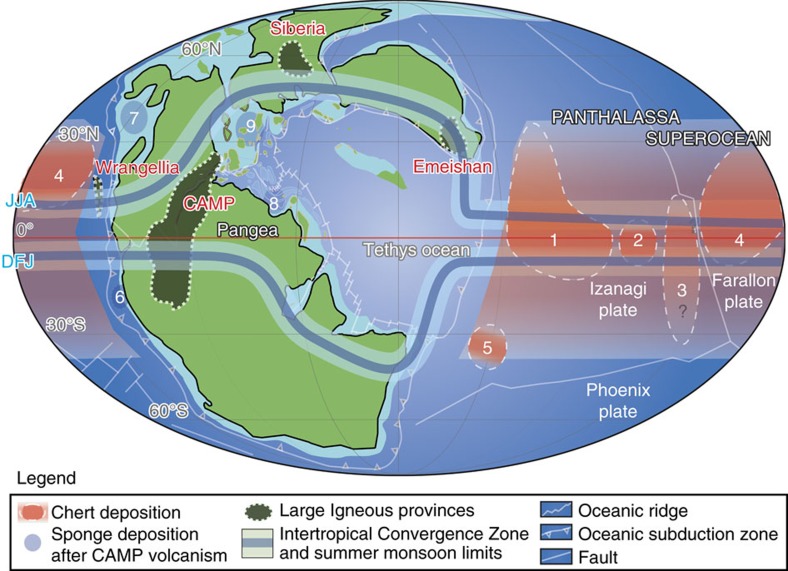
Palaeogeography in the early Mesozoic. Palaeogeographical map of Pangea and Panthalassa during the Triassic showing the potential distribution of bedded chert deposition (Orange shade) and the localities discussed in the text (dotted and blue areas): 1. Inuyama (central Japan); 2. Tsukumi (SW Japan); 3. Kamuikotan (N Japan); 4. Fraciscan (USA); 5. Waipapa (New Zealand); 6. Central Andes (Peru); 7. Gabbs Valley Range (USA); 8. High Atlas Mountains (Morocco); 9.Northern Calcareous Alps (Austria). The paleogeographic reconstruction is after Vrielynck and Bouysse^66^. The modelled positions of the ITCZ over ocean and the poleward limit of summer monsoon over land at JJA and DJF are from Kutzbach and Gallimore^42^.

**Figure 2:**
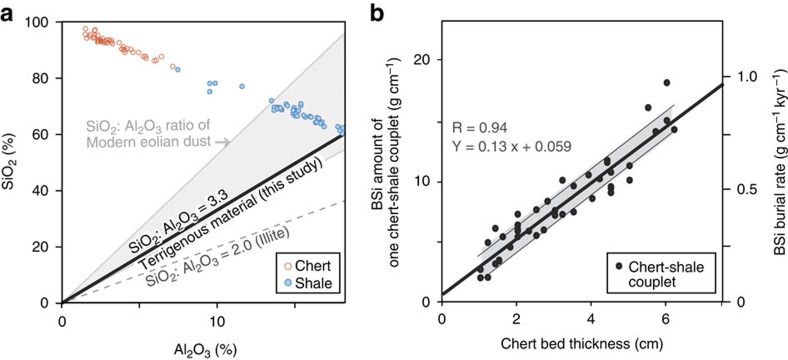
Major element composition and BSi burial fluxes of bedded cherts. (**a**) Relationship between SiO_2_ and Al_2_O_3_ (*r*=−0.98) of shale and chert beds and (**b**) positive relationship between chert bed thickness and accumulation amount of BSi per 1 cm^2^ per one chert-shale couplet with the corresponding BSi burial flux per one couplet on the right side of figure for the Lower-Middle Triassic bedded chert in the Inuyama area, central Japan. The SiO_2_:Al_2_O_3_ ratio of the sample with the smallest SiO_2_ content among all the analysed shale samples in this study was within the range of SiO_2_:Al_2_O_3_ ratio of the modern pelagic eolian dust^20^ (Grey shade). The dashed line is the SiO_2_:Al_2_O_3_ ratio of illite. Black line and grey shaded zone of (**b**) represent the regression line and standard deviation (1*σ*), respectively. Flux is calculated assuming one chert-shale couplet as a ∼20 kyr precession cycle.

**Figure 3:**
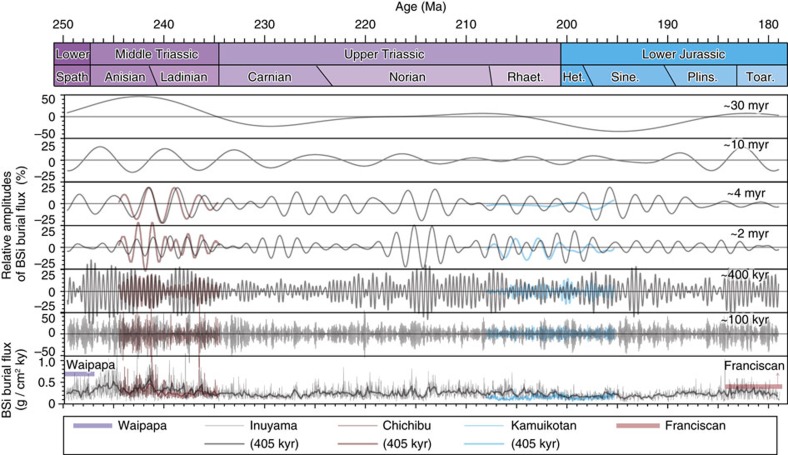
Orbital-scale changes in BSi burial fluxes of cherts. Temporal variations in the BSi flux during each precession cycle (thin line) and 405 kyr cycles (bold line) and the relative amplitude of the dominant orbital cycles (100 kyr to 30 Myr) of the early Mesozoic bedded chert sequence in the Inuyama area, central Japan. Also shown are the relative amplitudes of the dominant orbital cycles (100 kyr to 4 Myr) of the early Mesozoic bedded chert sequence in Chichibu and Kamiokotan areas, Japan, and average BSi flux in Waipapa area, New Zealand and Franciscan area, USA.

Lithologic and paleomagnetic studies of the bedded chert sequence in the Inuyama area, Mino Terrane, central Japan, suggest that plate motion translated the site of deposition translated from low latitudes during the Middle Triassic to low- to mid-latitudes during the Early Jurassic as a course of plate motion^17^,^18^,^19^ (Fig. 1). Clear negative correlations are observed between the abundance of SiO_2_ and terrigenous elements such as Al_2_O_3_, for individual chert and shale beds of the Middle Triassic bedded chert that were collected from the Inuyama area (Supplementary Figs 1 and 2, Supplementary Notes 1 and 2, and Fig. 2a). We assumed a SiO_2_/Al_2_O_3_ ratio of terrigenous material in the bedded chert to be constant at 3.3, which is that of a sample with the lowest SiO_2_ content in the analysed shales and is within the range of modern red clay^20^ (Fig. 2a). The calculated BSi content of one chert–shale couplet (equation (1); see Methods) shows a clear positive correlation with chert bed thickness (*r*=0.94; Fig. 2b). Based on previous work showing that each chert–shale couplet represents one climatic precession cycle (*ca*. 20 kyr), consistent with biostratigraphic and astrochronologic constraints^13^, the correlation between BSi content and chert bed thickness provides an estimate of the BSi burial flux for each precession cycle (equation (4); see Methods). The calculated BSi burial fluxes for bedded chert in Inuyama calculated per precession cycle and per 405 kyr cycle range from 0.14 to 1.4±0.064 g SiO_2_ cm^−2^ kyr^−1^ and from 0.21 to 0.62±0.058 g SiO_2_ cm^−2^ kyr^−1^, respectively, with an average value of 0.29±0.064 g SiO_2_ cm^−2^ kyr^−1^ during the Lower Triassic to Lower Jurassic (Fig. 3 and Supplementary Fig. 3).

## Discussion

During the early Mesozoic, radiolarian bedded cherts were widely deposited in the low–middle latitudes of both hemispheres across the Panthalassic superocean (Fig. 1). The calculated BSi burial fluxes for bedded chert in Inuyama are comparable to those for the same ages in other terranes, such as Chichibu (southwestern Japan), Kamuikotan (northern Japan), Franciscan (USA) and Waipapa (New Zealand) (Fig. 3 and Supplementary Note 3). Amplitudes of orbital-scale cycles of BSi burial fluxes between the bedded cherts in Inuyama and other sections are also comparable (Fig. 3). Phase differences of Myr-scale cycles exist between Inuyama and Kamuikotan sections. Such phase differences could have resulted from latitudinal differences in these depositional setting with lower upwelling intensity in the Kamuikotan section on the outside of the main equatorial upwelling region during periods of higher equatorial upwelling. This could be because the BSi burial flux and the relative amplitudes of orbitally induced cycles in the Kamuikotan section are lower than those of the Inuyama section^21^ (Figs 1 and 3). Nevertheless, the overall in-phase relations of Myr-scale cycles with similar amplitudes between Inuyama and Tsukumi sections^22^, deposited near the equator, support the idea that the BSi burial flux of the Inuyama bedded chert was representative of the overall Myr-scale BSi burial flux in the equatorial region (Fig. 3 and Supplementary Note 3).

In modern oceans, the major sinks for DSi are diatoms (6.3±3.6 Tmol Si per year) and siliceous sponges (3.6±3.7 Tmol Si per year)^9^. Most of these organisms live in coastal regions, continental margins (3.3±2.1 Tmol Si per year) and the Southern Ocean (2.2±1.0 Tmol Si per year), whereas the deep open ocean is a relatively minor contributor (<1.04±0.34 Tmol Si per year)^9^.

Before the ecological rise of diatoms during the Cretaceous, the radiolaria were the largest contributor to BSi accumulation^23^. Exceptional massive sponge spicule deposition occurred in shallow marine environments during the Carnian Pluvial Event (CPE; ∼230 Ma) and immediately after the end-Triassic mass extinction (ETE; ∼201.5 Ma), implying the possible temporal contribution of sponge DSi during the CPE and the aftermath of the ETE, although these sponge BSi fluxes are poorly constrained^24^,^25^ (Figs 1 and 3). Apart from the CPE and ETE, the overall lack of massive siliceous sponge deposition in the early Mesozoic implies that radiolarians in the pelagic ocean were the major oceanic DSi sink during most of the early Mesozoic.

Assuming that the BSi burial flux recorded in the Inuyama chert is representative of other low–middle latitude Panthalassic regions (*ca*. 1.2–2.1 × 10^8^ km^2^; Fig. 1) as above, the total BSi burial flux for low–middle latitude Panthalassan bedded cherts is estimated to be 2.3–7.5 × 10^17^ g SiO_2_ per kyr with an average of 5.3 × 10^17^ g SiO_2_ per kyr for a single 405 kyr cycle (Fig. 1). These values are ∼86% (∼22% to 470%) of the BSi burial flux in the modern global ocean over timescales of 10^4^ years (6.0±4.4 × 10^17^ g SiO_2_ per kyr)^9^. It is important to note that our estimated Mesozoic flux values tend to be underestimated, because the BSi burial in bedded cherts at higher latitudes is not included in the total. High latitudes BSi burial were probably not important because of the lack of isolated high latitude continents. This implies there was lower BSi productivity in the high latitudes than that of the modern ocean^9^,^26^. This is supported by the lack of known high-latitude bedded cherts of early Mesozoic age.

If, as we have shown, radiolarian BSi in chert was a major sink of DSi in the ocean during the early Mesozoic, then the fluctuations in global BSi burial flux recorded in bedded chert should have reflected variations in the input of DSi to the ocean on timescale longer than the oceanic residence time of DSi (<∼100 kyr^10^). At present, rivers account for >90% of total DSi input to the ocean, whereas contributions from eolian dust, seafloor weathering, and hydrothermal input are relatively minor^10^. As fluvial input of DSi is derived from terrestrial chemical weathering^27^,^28^,^29^, chemical weathering is the most plausible factor controlling the global BSi burial flux and bedded chert rhythms.

**Figure 4:**
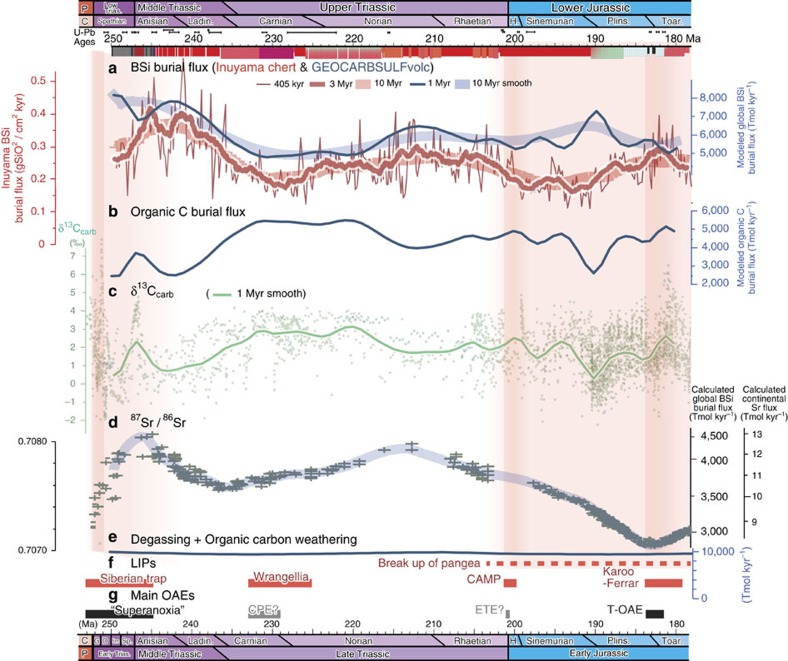
Comparison of BSi burial flux of cherts with GEOCARBSULFvolc model. Temporal variations in **a** BSi burial flux estimated from Inuyama bedded chert and modified GEOCARBSULFvolc, (**b**) organic carbon burial flux estimated from modified GEOCARBSULFvolc, (**c**) carbonate carbon isotope and (**d**) ^87^Sr/^86^Sr isotope record of seawater between 250 and 180 Ma^16^. *Y* axes on the right side of **d** are the calculated continental Sr flux and corresponding BSi burial flux (see Methods). Three Myr and 10 My smoothing window for BSi burial flux of Inuyama chert were chosen taking into account of the residence time of Sr in the ocean and the smoothing time step of GEOCARBSULFvolc^14^,^15^. (**e**) A combined flux of CO_2_ degassing and organic carbon weathering calculated from GEOCARBSULFvolc. Periods of (**f**) LIPs and (**g**) Main oceanic anoxic events (OAEs) are also shown. Possible regional OAEs associate with massive sponge deposition during CPE and ETE are shown in grey colour. Age model is revised according to Geologic Time Scale 2012 (ref. 65) and Ikeda and Tada^13^. The orange shade areas represent the periods of high hydrothermal flux intervals with higher probability on darker colour^52^,^67^,^68^.

To explore this hypothesis, we employed a biogeochemical model of carbon and sulfur, GEOCARBSULFvolc^14^,^15^, which uses the newly compiled isotope series of C, S and Sr (see Methods), and compared the calculated global silicate weathering rate with temporal fluctuations of BSi burial flux in the Inuyama bedded chert. As shown in Fig. 4a, the GEOCARBSULFvolc model demonstrates a similar temporal variation with BSi burial flux; at 10 Myr-resolution global BSi burial flux shows variations with peaks around 250–240 Ma and 210–200 Ma, and a minimum around 230–220 Ma, with a maximum amplitude of ∼20% (Fig. 4a; blue). Higher resolution results (1 Myr; blue thin line) also demonstrate the pattern similar to the ∼10 Myr cycle with relative amplitude of ∼20%. These model results are remarkable, because the modeled trend of silicate weathering rate is in good agreement with those of multimillion-year scale variations in the BSi burial flux observed of the Inuyama chert (Fig. 4a; red lines), despite the fact that GEOCARBSULFvolc does not take into account the direct effect of ∼10 Myr-scale (and possibly ∼30 Myr-scale) orbital forcing on climate. These long-term cycles are probably driven by the amplitude modulation of the ∼2 Myr eccentricity cycle due to the chaotic behavior of Solar planets that has similar periodicities^30^. If the CO_2_ degassing rate and the rate of oxidative weathering of organic matter were relatively constant over this interval, as documented in the models (Fig. 4e and Supplementary Fig. 4)^31^, the variations of silicate weathering might result mainly from *p*CO_2_ changes driven by the changes in organic carbon burial (Supplementary Fig. 4). The decoupling between the BSi burial flux record in the Inuyama chert and carbonate carbon isotope (δ^13^C_carb_) variations also supports this interpretation (Fig. 4a,d). Our result therefore implies that organic carbon burial and silicate weathering have changed in a complementary way over multi-million year timescales during most of the early Mesozoic (Fig. 4). It is important to note that the recent calculation of relative global CO_2_ degassing rate that takes into account total subduction zone length^32^ does not alter this feature (Supplementary Fig. 4).

The exceptions for coupling between our chert records and model are the Early-Middle Triassic and Early Jurassic Toarcian periods, characterized by oceanic anoxic events with massive volcanic activity of the Siberian Traps and Karoo–Ferrar Large Igneous Provinces (LIPs), respectively^33^,^34^,^35^. Such increased degassing events and their impact on the silicate weathering cannot be detected by the GEOCARBSULFvolc model due to the assumption of relatively stable degassing rate on multimillion-year scale, simply because it focuses on the long-term (> 1 Myr) geochemical cycle of carbon and sulfur, and degassing rate with Myr resolution. It is, however, important to note that the positive correlation between the δ^13^C_carb_ and BSi burial in chert during these periods (Fig. 4) could have resulted from a dynamic response of biogeochemical cycles to disturbance caused by the massive volcanic activity. Volcanism-induced global warming could have accelerated silicate weathering on land, and caused a resultant increase in the delivery of nutrients (for example, P and Si) to the oceans that promoted oceanic eutrophication and subsequent organic carbon burial via an enhanced biological productivity. As bottom waters became deoxygenated, preservation efficiency of organic carbon and regeneration efficiency of phosphorus by surface sediments could have also been enhanced^36^, resulting in the coupling of silicate weathering and organic carbon burial.

In addition to the GEOCARBSULFvolc model, calculated fluctuations in the chemical weathering rate and corresponding BSi burial flux at the 10 Myr-scale based on ^87^Sr/^86^Sr isotope (see Methods) are also consistent with the BSi burial flux of Inuyama chert^16^ (Fig. 4). As ^87^Sr/^86^Sr values vary positively with input of non-radiogenic mantle (or extra-terrestrial) Sr and vary inversely with input of continentally derived Sr, as usually assumed (see Methods), and the early Mesozoic was a period of relatively stable crustal construction^31^, the oceanic ^87^Sr/^86^Sr isotopic ratio was probably influenced more by global chemical weathering intensity (exception during the emplacement of the Early Triassic and Early Jurassic LIPs). During the Middle–Late Triassic, the ^87^Sr/^86^Sr isotopic ratio fluctuated significantly between 0.7070 and 0.7082, with positive peaks at about 245 and 215 Ma, and a negative trough at 235 Ma (Fig. 3). Quantification of the variation in chemical weathering intensity based on ^87^Sr/^86^Sr isotopic ratios indicates that the continental Sr flux varied from 1.0 to 1.3 × 10^13^ mol kyr^−1^ with relative amplitudes of ∼20% during the Middle–Late Triassic (Fig. 3). These variations in the continental Sr flux and their amplitudes are also in good agreement with Myr-scale variations in the BSi burial flux observed in the Inuyama bedded chert (Fig. 3).

The overall in-phase relationship between BSi burial flux for the Inuyama chert and silicate weathering rates calculated from the GEOCARBSULFvolc model, the early Mesozoic Sr isotope data and their parallel variations in amplitude supports the idea that the BSi burial flux, reconstructed from chert thickness, can be used as a semi-quantitative measure of global chemical weathering intensity during the early Mesozoic.

Large fluctuations (∼20 to 50%) in the BSi burial flux over the 100 kyr–30 Myr orbital cycles are observed in the Inuyama chert (Fig. 3). As the BSi burial flux is balanced with chemical weathering rates that can be approximated as a product of linear precipitation and Arrhenius temperature functions, the observed changes in the BSi burial flux could have been controlled by orbital-scale summer monsoon intensity changes^37^,^38^,^39^. However, these linear effects alone cannot explain the observed large relative amplitudes (∼20 to 50%) in the BSi flux of bedded chert, over the 100 kyr–30 Myr orbital cycles (Supplementary Note 4 and Supplementary Fig. 4), because its sinusoidal effect would be cancelled out between its maxima and minima over 20 kyr timescales with a nearly constant mean.

On these longer timescales, the emplacement of highly weatherable fresh continental flood basalts in the northern hemisphere, such as the Emeishan, Siberia, Wrangellia and CAMP LIPs, could have enhanced the hemispheric contrast of the chemical weathering rate (Fig. 1). Today, volcanic rocks exposed under monsoonal climate occupy only about 10% of land areas, but are responsible for more than 70% of the DSi fluxes to the ocean^40^. This areal fraction could have been larger in the early Mesozoic (Fig. 1).

The presence of the supercontinent Pangea would have further enhanced the susceptibility of silicate weathering rate to precession-scale dry–wet cycle through the latitudinal shift of the Intertropical Convergence Zone (ITCZ) and stronger monsoonal circulation, the so-called ‘mega-monsoon' driven by the large contrast in heat capacity between the supercontinent and the superocean^41^,^42^ (Fig. 1; Supplementary Note 5). The orbitally driven variations in the summer monsoon and chemical weathering intensity, have been reported from early Mesozoic sequences in low latitude regions^43^,^44^,^45^. Furthermore, precession-scale oscillations of the latitudinal shift of the ITCZ limit would have changed the areas of intense chemical weathering and the global chemical weathering (Fig. 1). In the regions near the precession-scale ITCZ limits, the dry extreme would have subjected the exposed rocks to less-weathered condition, whereas during wet extremes (*ca*. 10 kyr later), the intensive rainfall would have promoted weathering of less-weathered and highly weatherable fresh rocks up to 10 × faster^46^ (Supplementary Note 5). Subsequent formation of thick soil would have reduced the chemical weathering rate^40^. Eccentricity scale amplitude modulation of precession-induced oscillations of the ITCZ could have further amplified the changes in silicate weathering in the regions near the ITCZ limit. It is notable that the early Mesozoic LIPs and volcanic arcs were situated close to the northern limit of the latitudinal shift of the ITCZ (Fig. 1). All these features of early Mesozoic Pangea plausibly amplified the effects of orbital pacing of climate on chemical weathering and subsequent BSi burial.

On timescales longer than the residence time of carbon in the ocean and the atmosphere (∼500 kyr^47^), changes in organic carbon burial flux also modulated the global silicate weathering flux, as discussed to explain the similar BSi burial flux estimated by the Inuyama chert and the GEOCARBSULFvolc model (Fig. 4). Cenozoic δ^13^C_carb_ records show ∼2 Myr to ∼10 Myr cycles with negative values during the minima of these eccentricity cycles^48^. They interpreted this phasing to be caused by decreased (increased) organic carbon burial during the periods of stronger (weaker) summer monsoon intensity, consistent with our interpretation in terms of decoupling of silicate weathering and organic carbon burial (Fig. 4). Although the multimillion-year orbital-scale fluctuations in organic carbon burial are documented for Mesozoic terrestrial records in both low- and high-latitude regions and inferred from Jurassic-Cretaceous δ^13^C_carb_ records^44^,^49^,^50^, the mechanisms for this, such as oxidation/burial of organic matter by high frequency seasonally wildfires and shallow marine ventilation, remain unquantified^51^,^52^.

Our BSi burial record and GEOCARBSULFvolc model demonstrates that the imbalance in organic carbon cycle might have played a key role in the rate of silicate weathering on geologic timescales (Fig. 4a,b). Examining this hypothesis with proxy records and process-based models that can assess the hydrological cycle and its effect on terrestrial weathering with high enough spatial resolution would be critical to an improved understanding of the relationships between orbital forcing, climate, and biogeochemical cycles on geologic timescales.

## Methods

### Sampling methods and preparation

To estimate the BSi and terrigenous fluxes of bedded chert, we continuously sampling each chert and shale bed from bed number 110 to 156 (247.23±0.2 to 246.20±0.2 Ma) in the Lower Red Bedded Chert Unit of Ikeda *et al*.^5^ at section M^53^ in Inuyama area, whose total thickness is approximately 3 m (Supplementary Figs 1 and 2). A high-resolution age model for the Lower Triassic to Lower Jurassic bedded chert in the Inuyama area was constructed based on the cyclostratigraphy in conjunction with detailed radiolarian and conodont biostratigraphy and carbon isotope stratigraphy^13^. The astrochronology of the Inuyama bedded chert sequence was anchored at the end-Triassic radiolarian extinction interval^54^ of 201.4±0.2 Ma within bed 2,525 (ref. 13), where its age is constrained by the zircon U–Pb ages from the New York Canyon section (USA) and the Pucara section (Peru)^55^. We then extended by the 405 kyr-long eccentricity cycle of stable frequency with the ∼20 kyr precession signal as one chert-shale couplet for the high-resolution time scale^13^. This astrochronology is consistent with the available bio-chemostratigraphic age constraints^13^.

We selected this interval for continuous sampling of the individual chert and shale beds, because individual chert and shale beds in this interval are the thickest within the entire bedded chert sequence in the Inuyama area^5^ and individual chert beds are clearly distinguishable from adjacent shale beds. As there are intervals with more abundant Si-rich chert beds, such as the Upper Triassic recrystallized intervals (Supplementary Fig. 2), our estimation should be considered as the lower estimate. To conduct continuous sampling, we chose a transect where lateral changes in bed thickness, caused by tectonic deformation and diagenesis, is minimal^5^. Rocks were cut into slabs of ∼20 cm in vertical length, 10 cm in horizontal width and 3 cm in thickness using a power cutter on outcrop. The slab samples were sliced into square rods approximately 1 cm × 1 cm in horizontal dimensions and 0.2–8 cm in vertical dimension that represent a single bed of individual chert and shale.

### XRF measurements

To estimate the BSi and terrigenous material contents, the major elements composition was measured on lithium-tetraborate-fused glass discs using an instrument for X-ray fluorescence analysis (XRF; Philips PW-1480) equipped with a Rh tube at the Department of Earth and Planetary Science, the University of Tokyo. The measurement condition for the analysis of major elements was 40 kV, 60 mA for Rh tube with inside pressure of 1–2 Pa and temperature of 32±1 °C. The calibration lines for major elements were made following Yoshida and Takahashi^56^, using standard samples provided by the Geological Survey of Japan. A total of ten major elements (SiO_2_, TiO_2_, Al_2_O_3_, Fe_2_O_3_, MnO, MgO, CaO, Na_2_O, K_2_O and P_2_O_5_) for all samples analysed in this study are within 100±3%. The 2σ relative s.d. of reproducibility are within 0.2–0.4% for SiO_2_ and Al_2_O_3_, and better than 0.1% for the other eight elements.

Ninety-one samples, each representing a single chert and shale bed, were selected for XRF analysis. Samples of ∼1–10 g were crushed coarsely in a tungsten carbide mortar and then powdered in tungsten carbide mill for 2 min. Powdered samples of ∼0.6 g were heated at 100 °C in an oven for >4 h to remove absorbed water, then ignited at 1,000 °C in a furnace for 6 h to remove organic matter, water in chalcedony and clay minerals and CO_2_ in carbonate. Samples were stored in a desiccator for <4 h before making fused discs. Ignited powdered samples of 0.4000±0.0002, g were mixed with a Li_2_B_4_O_7_ flux of ∼4.000±0.002 g with the ratio of 1:10,000 and heated at 1,000 °C for 5 min to prepare fused discs^56^.

### Estimation of BSi and terrigenous material contents

To estimate the BSi content from the major element concentrations of bedded chert, it is necessary to know the SiO_2_ and Al_2_O_3_ contents of the terrigenous material in bedded chert. As BSi in bedded chert tends to have migrated from layers with lower Si contents to adjacent layers with higher Si contents during the diagenetic silica phase transformation from opal-CT to quartz^57^, SiO_2_ content in shale tends lowered during the diagenetic process. Consequently, the lowest SiO_2_ shale is plausibly representative of the SiO_2_ content of terrigenous material in bedded chert. The SiO_2_ and Al_2_O_3_ contents and the SiO_2_/Al_2_O_3_ ratio of the lowest SiO_2_ shale of all samples analysed are 62.4%, 18.9% and 3.30, respectively (Fig. 2a). These values could overestimate the SiO_2_ content and SiO_2_/Al_2_O_3_ ratio and underestimate the Al_2_O_3_ content of terrigenous material, considering the possibility that some BSi may still even remains in the shale sample with the smallest SiO_2_ content. Nevertheless, the SiO_2_/Al_2_O_3_ ratio of 3.3 is within the range between 3 and 5.5 for the modern red clay and smaller than the average value of 4.5 (ref. 20). In this study, we assume that the Al_2_O_3_ content and SiO_2_/Al_2_O_3_ ratio of the terrigenous material in bedded chert are 18.9% and 3.30, respectively, with the caveat that it may underestimate BSi content (Fig. 2a).

Based on these assumptions, we estimated the BSi and terrigenous material contents, as follows;


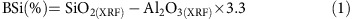



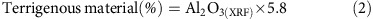


where SiO_2 (XRF)_ and Al_2_O_3 (XRF)_ are the SiO_2_ and Al_2_O_3_ contents of a sample determined by XRF, respectively; 3.3 and 5.8 are constants that represent the SiO_2_/Al_2_O_3_ ratio and 100/Al_2_O_3_ (%) of terrigenous material in chert, respectively (Fig. 2a).

Although diagenetic SiO_2_ migration occurs from layers with lower Si content to adjacent layers with higher Si content during the silica phase transformation^57^, SiO_2_ could not have been exported across more than one chert-shale couplet, because each chert bed was a sink for the SiO_2_ of the adjacent shale bed. To control for the possible diagenetic enhancement of the differences in SiO_2_ between a chert bed and adjacent shale bed, we calculated the accumulation amounts of BSi and terrigenous material by per unit area per one chert-shale couplet. We defined one chert-shale couplet as the interval from the centre of a shale bed to the centre of the next shale bed.

We calculated the accumulation amounts of BSi and terrigenous material in one chert-shale couplet corresponding to the chert bed number ‘*n*' [*X*_*n*, couplet_] (g cm^−2^) as follows;


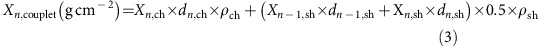


where *X* is the amount of BSi or terrigenous material deposited per unit area (g cm^−2^), *d* is the thickness of a chert or shale bed (in cm) and *ρ* is average dry bulk density (g cm^−3^) for chert and shale beds assumed to be 2.46g cm^−3^ for chert and 1.69 g cm^−3^ for shale^6^. The suffix, such as ch, sh, couplet and *n* represents chert, shale, a couplet and bed number *n*, respectively.

### Estimation of BSi flux for Inuyama bedded chert

The BSi and terrigenous fluxes for the bedded chert were calculated as the accumulation amounts of BSi and terrigenous material per couplet per unit area divided by duration of deposition for one chert-shale couplet. Based on the clear positive correlations between accumulation amount of BSi for one chert-shale couplet and chert bed thickness (*r*=0.94; Fig. 2b), we derived an equation to estimate the accumulation amount of BSi for a chert bed of bed number *n* (*d*_*n*,ch_), as follows;


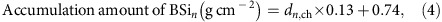


where 0.13 and 0.74 represent a slope and *y*-intercept of the regression line between accumulation amount of BSi and chert bed thickness, respectively (Fig. 2b). The estimation error for BSi based on this regression is±1.1 g cm^−2^.

As shown by Ikeda *et al*.^5^, the amount of time represented by each chert-shale couplet corresponds to the duration of a single climatic precession cycle. Strictly speaking, the duration of a precession cycle varies from 15 to 23 kyr with a s.d. of 2 kyr during the Triassic^58^. Thus, the relative errors for the BSi and terrigenous material burial fluxes for one precession cycle are ±10%. The 405 kyr eccentricity cycle has also been identified in the chert bed thickness variations^5^,^13^. The period of the 405 kyr eccentricity cycle is stable and nearly constant in duration based on astronomical theory^59^ and therefore we also calculated the burial rate of each component for every 405 kyr cycle with relative errors of <0.05% for the duration of this cycle^59^.

### Estimation of BSi in chert as Si sink

To estimate the contribution of bedded chert in the biogeochemical silica cycle during the Mesozoic, we examined the worldwide distribution of the early Mesozoic bedded cherts, widely found in Mesozoic circum-Pacific accretionary complexes^60^ (Fig. 1). The paleolatitudes of bedded cherts give us the minimum range of their latitudinal distribution (Fig. 1). Although bedded chert distribution could have extended to higher latitudes, no high latitude pelagic records are presently known.

We estimate the global BSi burial flux in the form of bedded chert during the Early Triassic to Early Jurassic and compiled the BSi burial fluxes for all bedded chert sequences of the area other than the Inuyama area. The average BSi burial flux for a bedded chert sequence was obtained from the thickness of the stratigraphic interval, the average dry bulk densities of chert and shale beds and the total duration of the interval, with the assumption that a positive correlation between chert bed thickness and BSi amount for a chert-shale couplet, as is seen in the Inuyama bedded chert (equation 4).

### Amplitudes of the eccentricity-scale BSi burial flux

To estimate the impact of orbital forcing on the BSi burial flux for the bedded chert, we estimated the amplitudes of orbital-scale variations in the BSi burial flux for the early Mesozoic bedded chert in the Inuyama area. To detect the relative amplitudes of changes in BSi burial rates in these dominant periodicities during the Early Triassic to Early Jurassic, we used band-pass filters selected to capture the dominant spectral powers (Fig. 3). We performed band-pass filtering using a Gauss algorithm in the free software AnalySeries 2.0.4.2 (ref. 61) to extract these dominant spectral components.

### Modified GEOCARBSULFvolc model

To estimate the global silicate weathering rate, we employed the long-term carbon and sulfur geochemical model, GEOCARBSULFvolc, which is an improved version of the original model developed by Berner^62^. In Royer *et al*.^15^, several improvements on the forcings, such as the land-area fraction that actually undergoes chemical weathering, sensitivity of runoff to changes in global mean surface temperature and global-mean land surface temperature have been made based on the results obtained by GEOCLIM^62^,^63^, a global carbon-alkalinity cycle model coupled with AGCM^15^. K.O. wrote the FORTRAN scripts, which can reproduce the reference run presented in Royer *et al*.^15^. We assumed the initial condition of Royer *et al*.^15^ at *t*=570 Ma (where *t* is age) and ran GEOCARBSULFvolc with their input arrays until *t*=250 Ma. For our target interval (*t*=250–180 Ma), we used the newly compiled isotope series of carbon, sulfur and strontium (Supplementary Data 1; see below), which enable us to obtain a multimillion-year timescale global silicate weathering rate. The time step was set at 1 Myr in this study. Other parameter values were set at the standard value of Royer *et al*.^15^.

In the GEOCARBSULFvolc model, isotope series are pivotal for assessing the variations of carbon and sulfur cycles. In the original models^15^, outputs are shown as 10 Myr averaged value, because some of the input parameters are not well constrained on shorter timescales. Nonetheless, the variations of carbon and sulfur geochemical cycles on a million-year timescale can be discussed with a reasonable confidence if we adopt the appropriate composite time series for isotopic data. For the sake of a better isotope time series, we conducted a literature survey for carbon and sulfur isotopic data slightly modified to the recent geologic time scale^65^. The carbon and sulfur isotope records were fitted with a smoothed spline function in R with a *smooth.spline* command and a smoothing parameter (0.75 and 1.25 for the string of carbon and sulfur isotopes, respectively). For strontium isotope data, we adopted the compilation by McArthur *et al*.^16^. For sulfur isotope series, our input array is similar to that of Royer *et al*.^15^. On the other hand, carbon isotope data used in this study reflects the multimillion-year timescale variations (such as ±1 per-mil variations in the Early Triassic and Jurassic) (Fig. 4). Strontium isotope data compiled by McArthur *et al*.^16^ is characterized by a peak at 250–240 Ma and at ∼210 Ma, and a secular decrease thereafter. As ^87^Sr/^86^Sr reflects the global fraction of volcanic rock weathering (it is faster than non-volcanic rock weathering), this improvement affects the weatherability on land and atmospheric CO_2_ level.

### ^87^Sr/^86^Sr ratio as measure for chemical weathering

The ^87^Sr/^86^Sr signature of seawater also reflects changes in the relative contribution between two major strontium sources to the ocean and their isotope ratios. The two major Sr sources are the riverine input and the hydrothermal input at mid-ocean ridges. Riverine ^87^Sr/^86^Sr is characterized by radiogenic Sr with relatively high ^87^Sr/^86^Sr isotopic ratio due to weathering of old continental crust, whereas hydrothermal ^87^Sr/^86^Sr is characterized by relatively low ^87^Sr/^86^Sr isotopic ratio^16^. If we can assume that the Sr flux of hydrothermal activity was relatively constant, and that Sr isotopic values of the riverine and hydrothermal inputs were constant, respectively, the ^87^Sr/^86^Sr isotopic ratio of the ocean should be controlled by the riverine input. The Early Triassic to Early Jurassic is considered to be relatively stable with respect to hydrothermal activity based on the hypothesis that 10^7^ to 10^8^ year-scale eustatic sea level changes are due primarily to changing ridge volume^31^. Exceptions are the Early–Middle Triassic and the latest Triassic to Early Jurassic intervals, which are known as the period of the prolonged Siberian Trap activity and the break-up of Pangea associated with massive volcanic activity in CAMP and Karoo–Ferrar LIPs, suggesting significant increase in the hydrothermal activity^33^,^34^,^35^. In addition, these continental magmatic activities may have increased hydrothermal Sr input and decreased Sr isotopic values of riverine input. The latter may be due to increased contribution of chemical weathering of less radiogenic basalt^16^.

To quantitatively estimate the variations in the global chemical weathering intensity from the seawater ^87^Sr/^86^Sr isotopic ratio, the hydrothermal flux, the isotopic values of hydrothermal sources and continental sources were assumed as constant and similar to the modern values of 0.92 × 10^16^ mol Myr^−1^, 0.7025 and 0.7114, respectively^16^,^37^. To estimate the corresponding global BSi burial flux, it is assumed to be in the same proportion to continental Sr input and modern global BSi burial flux as 8,300 Tmol kyr^−1^ (ref. 14). To compare the BSi burial flux and ^87^Sr/^86^Sr isotopic ratio, 3 Myr smoothing window for BSi burial record of the Inuyama bedded chert was chosen taking into account the residence time of Sr in the ocean.

### A simple weathering model for eccentricity-scale weathering

Regional chemical weathering rates can be approximated as a function of runoff and Arrhenius temperature functions based on modern observations^39^. We use a function that quantitatively describes weathering flux of SiO_2_ (*Q*i,w) as a coupled product of linear precipitation *P* and Arrhenius temperature functions^38^,^39^,


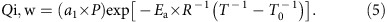


*R* is the gas ideal constant, *E*_a_ is an apparent activation energy and *T*_0_ is a reference temperature. The pre-exponential term on the right-hand side of equation (5) assumes a linear correlation between precipitation and SiO_2_ flux where *a*_1_ is the slope. The effect of temperature *T* on weathering rates involving the silicate dissolution is commonly described by an Arrhenius relationship. The Arrhenius relationship is derived for kinetics of simple well-characterized chemical reactions. The exponential temperature term in equation (5) is equivalent to the Arrhenius relationship but describes the variation in fluxes as a function of the difference between *T* and a reference temperature *T*_0_ (ref. 39).

Equation (5) can be solved numerically for the values of *a*_1_ and *E*_a_ maximizing the correlation between the predicted and measured watershed fluxes as functions of temperature and precipitation. Solutions to equation (5) require the selection of a reference temperature (*T*_0_), which was chosen to be 5 °C, near the mean of the temperature data of White and Blum^39^. In fitting equation (5) to the watershed data, precipitation and temperature are treated as independent variables with respect to each other. The correlation between *P* and *T* has an *r*^2^=0.08, suggesting that this is a valid assumption^39^. The resulting fit of the SiO_2_ data to the coupled precipitation-temperature function (equation (5)) produces values of *a*_1_=0.456 and *E*_a_=59.4 kJ mol^−1^ (ref. 39).

Relative amplitudes of orbital-scale chemical weathering rate during the Mesozoic can be calculated by using model-based changes in temperature and precipitation. The GCM model simulated changes in temperature and precipitation at three locations on the southern continent of idealized supercontinent Pangea during Triassic^41^. The regions are the southern tropical interior, the southern low-latitude coast and the southern middle-latitude interior (Supplementary Fig. 5). According to the GCM model results by Kutzbach^41^, the functions for *P* and *T* in these regions are as follows:


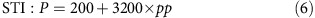



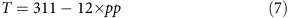



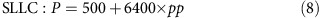



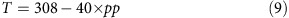



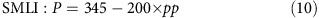



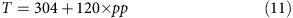


where *pp* is the precession parameter. With moderate eccentricity of the earth's orbit (0.025), the enhanced seasonality of insolation in the hemisphere with summertime perihelion (wintertime aphelion) produces increases of precipitation along southern tropical interior and southern low-latitude coast of about 20% and 40%, and changes in temperature of 0.3 °C and 1 °C, respectively^41^ (Supplementary Table 1). In contrast, the southern middle-latitude interior might have only very small changes in precipitation of about 1.6% but large changes in temperature. Summers could be 6 °C (1% for absolute temperature) warmer when perihelion is in summer than when it is in winter^41^ (Supplementary Table 1). The corresponding precession-scale climatic changes in the Northern Hemisphere would be out of phase with the changes in the Southern Hemisphere.

Orbital parameters of last 10 Myr^59^ are used to estimate GCM-based changes in temperature and precipitation for the relative amplitude of eccentricity-scale chemical weathering rates, described above. As all available solutions have large uncertainty before 50 Ma due to the chaotic behavior of Solar system^59^, orbital parameters of Laskar *et al*.^59^ are therefore used only as examples of the potential variability.

### Data availability

The data that support the findings of this study are available from the corresponding author on request.

## 

## Additional information

**How to cite this article:** Ikeda, M. *et al*. Astronomical pacing of the global silica cycle recorded in Mesozoic bedded cherts. *Nat. Commun.* **8**, 15532 doi: 10.1038/ncomms15532 (2017).

**Publisher's note:** Springer Nature remains neutral with regard to jurisdictional claims in published maps and institutional affiliations.

## Supplementary Material

Supplementary Information Supplementary Figures, Supplementary Table, Supplementary Notes and Supplementary References

Supplementary Data 1 d13C, d34S, and 87Sr/86Sr isotopes of the early Mesozoic carbonate rocks
