# Supplementary material for: Astronomical pacing of the global silica cycle recorded in Mesozoic bedded cherts
Source: Nat Commun. 2017 Jun 7;8:15532. doi: 10.1038/ncomms15532 (PMC5467233; doi:10.1038/ncomms15532)
Supplement: Supplementary Information — Supplementary Figures, Supplementary Table, Supplementary Notes and Supplementary References [file ncomms15532-s1.pdf]

Supplementary Note 1. Major element contents

Major elements compositions were analyzed for ninety-one samples to estimate the BSi and terrigenous contents. The analyzed samples include two rock types, chert and shale. In all samples analyzed, SiO_2 is a major component (60.4 % to 97.9 %), followed by Al_2O_3 (1.5 % to 18.1 %) (Fig. 2). The SiO_2 contents in chert beds (80.5 % to 97.9 %) were higher than in shale beds (60.4 % to 80.1%) (Fig. 2).

Correlations between elements were examined for all the elements analyzed in order to estimate BSi content from the major element composition of samples. SiO_2 content is negatively correlated with contents of other elements, especially Al_2O_3, TiO_2, Fe_2O_3, MgO, and K_2O (r > -0.90). Al_2O_3, TiO_2, Fe_2O_3, MgO, and K_2O contents are positively correlated with each other (r > 0.90). Because Al is the major component of terrigenous materials that are dominantly contained in aluminosilicates, and is considered as one of the most immobile elements in the surface environment[1], it is reasonable to regard Al as a representative element of terrigenous materials. Consequently, elements that show high positive correlation with Al are also regarded as being held mostly in terrigenous materials. Thus, these elements are named the terrigenous elements. Lower but still positive correlations are observed between Al_2O_3 and MnO, P_2O_5, and

CaO (0.70 < r < 0.90). Relatively low correlation coefficients may reflect association of parts of these elements with authigenic phases, such as Mn-oxides and apatite, in addition to their association with terrigenous materials[2]. There is no clear correlation between Na_2O and Al_2O_3 (r = 0.25), although Na is also considered to be held dominantly in aluminosilicates[1]. This is probably because Na_2O is easily leached during surface weathering and vaporized by ignition during sample preparation[1]. Because contents of MnO, P_2O_5, CaO, and Na_2O are less than 1% in samples analysed, these elements will not be discussed in this study.

Supplementary Note 2. Estimation of BSi and terrigenous material contents

To estimate the BSi content from the major element contents of bedded chert, it is necessary to know SiO_2 and Al_2O_3 contents of the terrigenous material in bedded chert. Previous studies on bedded chert assumed that SiO_2 and Al_2O_3 contents of terrigenous material as 52.8% and 26.1%, respectively, with SiO_2/Al_2O_3 ratio of 2.02, which based on the chemical composition of illite[3, 4, 5] (Fig. 2A). However, the terrigenous material accumulated in the modern pelagic ocean contains not only illite, but also quartz and feldspars with their contents being 20 to 40% and 15 to 30%, respectively, based on the mineral compositional analysis of

pelagic red clay[6, 7]. Therefore, SiO_2/Al_2O_3 ratio of terrigenous material accumulated in the modern pelagic ocean is 3 to 4.5 based on the major element chemical analysis of pelagic red clay[6, 7] (Fig. 2A). Thus, the terrigenous material in bedded chert is probably more SiO_2 rich, and its SiO_2/Al_2O_3 ratio should be larger than previously estimated.

Assuming the smallest SiO_2 content among all the analyzed shale samples should be close to the SiO_2 content of terrigenous material in bedded chert, the calculated BSi contents in individual chert and shale beds range from 75 to 93% and 0 to 75% with average values of 81% and 20%, respectively. The standard deviations of calculated BSi contents of the individual chert and shale beds are 7.8% and 14% with a relative standard deviation of 9.6% and 70%, respectively (Fig. 2A). The accumulation amounts of BSi and terrigenous material per one chert-shale couplet per unit area range from 2.5 to 14 g cm^{-2} and from 0.5 to 5.1 g cm^{-2} with average values of 6.8 g cm^{-2} and 3.0 g cm^{-2}, respectively (Fig. 2B).

Supplementary Note 3.Estimation of BSi flux in cherts as oceanic Si sink in the early Mesozoic Panthalassa superocean

To support the significant contribution of bedded chert for the biogeochemical silica cycle

in the Mesozoic ocean, we examined the worldwide distribution of the early Mesozoic bedded cherts. The paleolatitude estimation at the time of deposition of the Middle and Upper Triassic bedded chert in the Inuyama area suggests low latitudes (5.6° ± 2.2°) and northern low to middle latitudes (29.5°N ± 17.4°) of western Panthalassa[8, 9, 10] (Fig. 1). The Middle Triassic to Middle Jurassic bedded chert in the low latitude (2.1◦ ± 5.2◦S) of the eastern Panthalassa was found in Tsukumi section, Shakumasan Group of the Chichibu Terrane, Kyushu, southwestern Japan[11] (Fig. 1 and supplementary Fig. 1). The Upper Triassic to Lower Cretaceous bedded chert of the western Panthalassa with no paleolatitude data was found in the Pisenaizawa section, Kamuikotan Terrane, Hokkaido, northern Japan[12, 13] (Fig. 1 and Supplementary Fig. 1). The paleolatitude of the Pisenaizawa section can be inferred as low latitude on the basis of the nearby Upper Cretaceous fore-arc basin sequence in the low latitude[14] (16.7◦ +11.0/-9.8◦N) and plate motion direction of the Izanagi Plate[15]. The distance that these terranes traveled prior to accretion is not known with certainty, but a rough estimate of 2100–4200, 2400–4800, 3000–6000 km can be made on the basis of approximately ~70-Myr (Early Triassic– Early Jurassic), ~80-Myr (Early Triassic–Middle Jurassic), and ~100-Myr (Late Triassic– Early Cretaceous) travel history, respectively, and a rough convergence rate of 3–6 cm yr^{-1} [15]. The Lower Triassic

bedded chert deposited at the southern middle latitude (34°S ± 8°) of western Panthalassa was found in Waiheke island of the Waipapa composite Terrane, New Zealand[16] (Fig. 1). The Lower Jurassic to Lower Cretaceous bedded chert deposited at low latitude (0 ± 2°, 1 ± 2°, 2 ± 4°)[17] to the middle latitude (32°N ± 8°)[18] of western Panthalassa was found in the Franciscan Terrane, North America (Fig. 1). Thus, it can be concluded that bedded chert was widely deposited at least in the low to middle latitude in the both hemispheres of the eastern and western Panthalassa during the Early Triassic to Early Jurassic (Fig. 1).

The superocean Panthalassa comprised an area of 80–90% in area of the global ocean during the Early Triassic to Early Jurassic (Fig. 1)[19]. Although its distribution could have been extended to higher latitudes, there are no high latitude pelagic records available at this moment. Assuming that bedded chert covered the area of the low latitude Panthalassa between 30°±10° S and N, the depositional area of bedded chert would have occupied at least approximately 40 to 60 % (~1.2-2.1 x 10^8 km^2) of the global ocean during the Early Triassic to Early Jurassic (Fig. 1).

We also compiled the BSi burial fluxes for bedded chert sequences of the area other than the Inuyama area to support the claim that the BSi records of the Inuyama bedded chert is

representative of the low-mid latitudes of Panthalassa (Fig. 3). The average BSi burial fluxes for the Middle Triassic bedded chert of the equatorial western Panthalassa in the Tsukumi section are 0.25 to 0.34 g $cm^{-2}\,kyr^{-1}$ with average value of 0.29 g $cm^{-2}\,kyr^{-1}$ [20,21,22]. The average BSi burial flux for the Upper Triassic to Lower Jurassic bedded chert of the central Panthalassa in the Pisenaizawa section ranges from 0.12 to 0.19 g $cm^{-2}\,kyr^{-1}$ with an average value of 0.15 g $cm^{-2}\,kyr^{-1}$ [12,13]. The average BSi burial flux for the Lower Jurassic bedded chert deposited in the low latitude of eastern Panthalassa was >0.40 g $cm^{-2}\,kyr^{-1}$ (at Franciscan Terrane, western North America; Murchey, 1984, Hagstrum et al., 1993). These Myr-scale BSi burial fluxes are the same order as those for the Inuyama bedded chert (Fig. 2; 0.18 to 0.39 g cm^{-2} kyr^{-1} with average value of 0.29 g cm^{-2} kyr^{-1}). Phase differences of Myr-scale cycles between the Inuyama and Pisenaizawa sections might have resulted from latitudinal difference with probably lower upwelling intensity of Pisenaizawa section on the outside of main equatorial upwelling region during periods of higher equatorial upwelling (Fig. 1). The intensified equatorial upwelling could have enhanced spatial variations of BSi burial, which makes anti-phase relationship between the inside and outside of upwelling regions. This interpretation is consistent with the lower BSi burial flux and the relative amplitudes of orbital cycles in Pisenaizawa section than

those of Inuyama section (Fig. 3).

The BSi burial fluxes for the Lower Triassic bedded chert in the southeastern Panthalassa range from 0.61 to 0.76 g cm^{-2} kyr^{-1} with an average value of 0.66 g cm^{-2} kyr^{-1} (Waiheke section, Waipapa Terrane, New Zealand[23]. This estimate is much larger than that of Inuyama area. However, in the Inuyama area, Sakuma et al.[24] reported the Smithian-Spathian siliceous claystone sequence with a linear sedimentation rate several times higher than that of the overlain bedded chert sequence, suggesting a much higher BSi burial flux during the Smithian-Spathian, although the BSi contents and BSi burial flux of Smithian/Spathian sequence has never been estimated. Thus, it can be concluded that the BSi burial flux of the Lower Triassic sequences in the Inuyama and Waiheke sections would be same order.

Supplementary Note 4. Simple weathering model for orbital-scale variations in chemical weathering

Regional chemical weathering rates can be approximated as a function of runoff and Arrhenius temperature functions based on the modern observations[25, 26, 27, 28] (see Methods). The results show small amplitudes of <0.1% for local weathering flux, and <0.01 % for global

weathering flux on timescales longer than 100 kyr (Supplementary Fig. 6; Supplementary Table 1). These small effects can be caused by the negligible effect of climatic precession on insolation on timescales longer than one precession cycle, because effect of climatic precession would be cancelled out between its maxima and minima due to its sine-wave pattern, and between hemispheres. Half-precession signal with relative amplitudes of <0.01 % can be recognized in the results of bipolar regions because of the combination of anti-phased climatic precession signal amplified by exponential temperature term.

Supplementary Note 5. Orbital-scale ITCZ shift as a potential amplifier mechanism of orbital forcing

Although the calculated relative amplitudes of orbital-scale chemical weathering rates by the simple weathering model is negligible, chemical weathering is a highly non-linear system. In this section, we discuss the possible non-linear amplification mechanism of the global chemical weathering intensity through the orbitally-forced summer monsoon intensity changes during the early Mesozoic.

GCM results demonstrated that the existence of the supercontinent Pangea could have

enhanced the latitudinal shift of the Inter-Tropical Convergence Zone (ITCZ)[29]. The poleward directed summer monsoon flow causes a shift of the ITCZ from a near equatorial hinge point along the west coast of Pangaea to mid-latitude at the westernmost coast of the Tethys Sea[29] (Fig. 1). Further to the east, the moisture-bearing summer monsoon flow would penetrate about 1,000 km inland from the Tethys Sea, and would reach up to 60° latitude along the east coasts of Pangaea[29] (Fig. 1).

The areas of northern and southern limits of the annual shift of ITCZ would have changed in the orbital scale[30] (Fig. 1). In the course of a precession cycle, both of the northern and southern limits of the annual ITCZ shift northward in northern hemisphere summer at perihelion, and shift southward in northern hemisphere summer at aphelion[30]. This latitudinal shift of ITCZ limits would have changed the distribution of precipitation and areas of the humid region, and hence the global chemical weathering intensity.

In addition, precession-scale latitudinal shift of the ITCZ limits could make the transitional regions, which are located out of the range of the annual shift of ITCZ in summer hemisphere at ahelion, and are located within the range of the annual shift of ITCZ in summer hemisphere at perihelion, mainly in eastern Pangea[30]. In these transitional regions, the contrast

of variations in chemical weathering rate and precipitation in a precession cycle would have been quite large, due to the effect of time dependency of chemical weathering rates, which is related to the saturation of chemical weathering rates with time, may enhance the contrast of chemical weathering rates on timescale of precession cycle[31]. This effect on chemical weathering rate (R; mol m^{-2} s^{-1}) was described by the power function of time t as R = 3.1 x (10^{-13}) $t^{-0.61}$ [31].

The amplitude of the latitudinal shift of the northern and southern limits of the ITCZ in a precession cycle could be modulated by the eccentricity because eccentricity cycle modulates the amplitude of precession cycle[32]. Hence, the eccentricity cycle could have modulated the area affected by the time dependency of chemical weathering rates. In addition, the enhanced seasonal contrast during maximum eccentricity promotes the exposed rocks to have been extremely less weathered during the drier dry period of one precession cycle, and the exposed extremely less weathered rocks would have been extremely easily weathered during the wetter wet period of one precession cycle due to the effect of time dependency of silicate weathering rates[31]. This effect would have enhanced the chemical weathering at regions near the northern and southern limits of the annual shift of ITCZ, and would have contributed to variations in the

global chemical weathering intensity. In fact, the effect of the time dependency of chemical weathering rates is qualitatively consistent with the variations in the chemical weathering intensity in the German basin, which show the abrupt increases in chemical weathering intensity during the periods of lake level rises, and subsequent gradual decreases in chemical weathering intensity during the periods of lake level drops in association with eccentricity cycles[33].

Significant amplitudes of orbital-scale changes in BSi burial flux of the early Mesozoic bedded chert should be explained by these amplifier mechanisms of orbital forcing within the Earth surface processes. To quantify the magnitude of the latitudinal shift of the ITCZ and the time dependency of chemical weathering rates driven by the orbitally-forced summer megamonsoon intensity changes, the variations in the latitudinal shift of the ITCZ and spatial variations of precipitation and chemical weathering rates on orbital timescales need to be estimated by terrestrial paleoclimatic records of the precipitation and chemical weathering intensity, and by theoretical study using climate models on atmospheric circulation and global geochemical cycles, including chemical weathering.

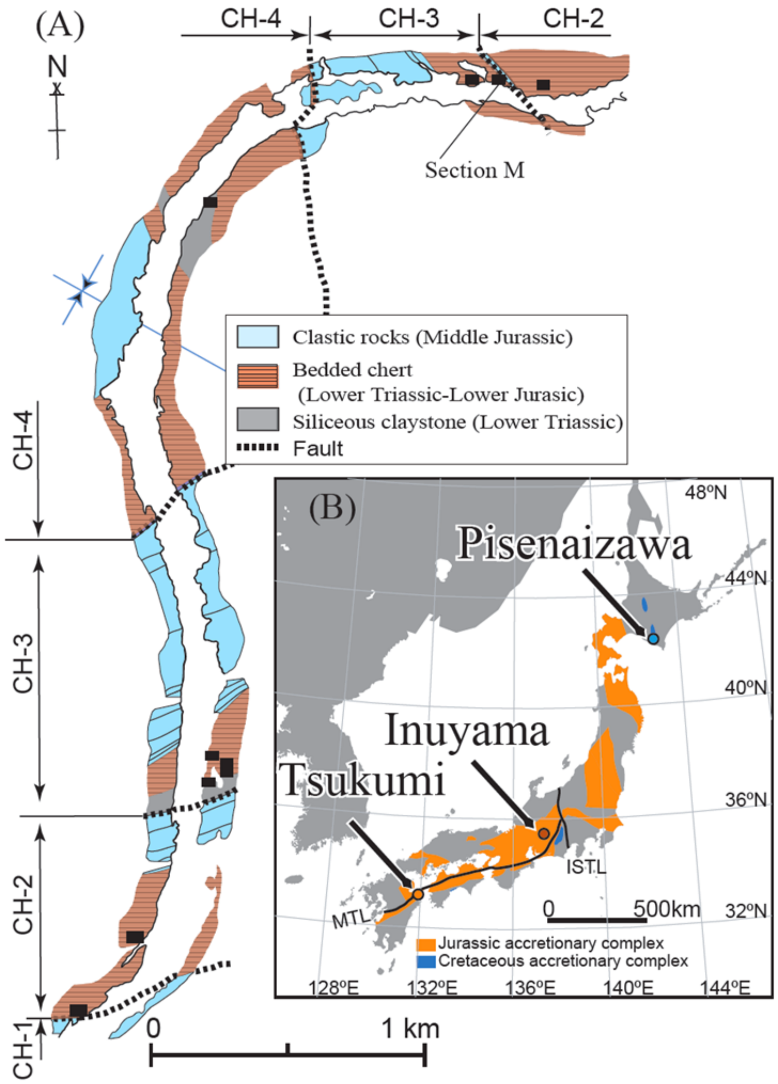

Supplementary Figure 1. Geologic map of the study area. (A) geologic map of the section M of the Inuyama area modified after Matsuda and Isozaki[76] and Ikeda and Tada[77], and (B) location map (inset) of the Inuyama area (Mino Terrane), Tsukumi section (Chichibu Terrane), and Pisenaizawa section (Kamuikotan Terrane).

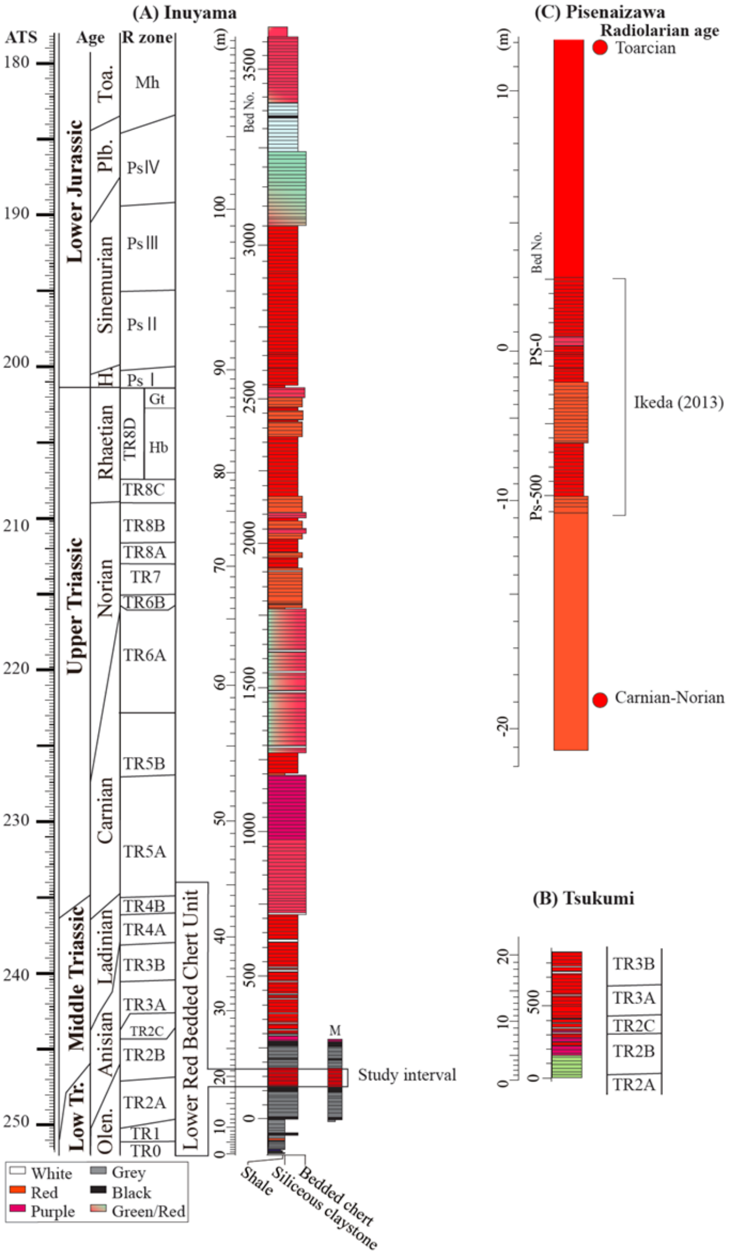

Supplementary Figure 2. **Lithostratigraphy of the Lower Triassic to Lower Jurassic bedded chert sequences.** Rock types are from Sugiyama (1997), Ikeda et al., (2010), Sakuma et al., (2012), and Ikeda (2013). Radiolarian zones (R zones) are based on Hori (1990), Sugiyama (1997), and Carter and Hori (2005). Astronomical time scale (ATS) is from Ikeda and

Tada (2014). Also shown are section M as the interval for chemical analysis.

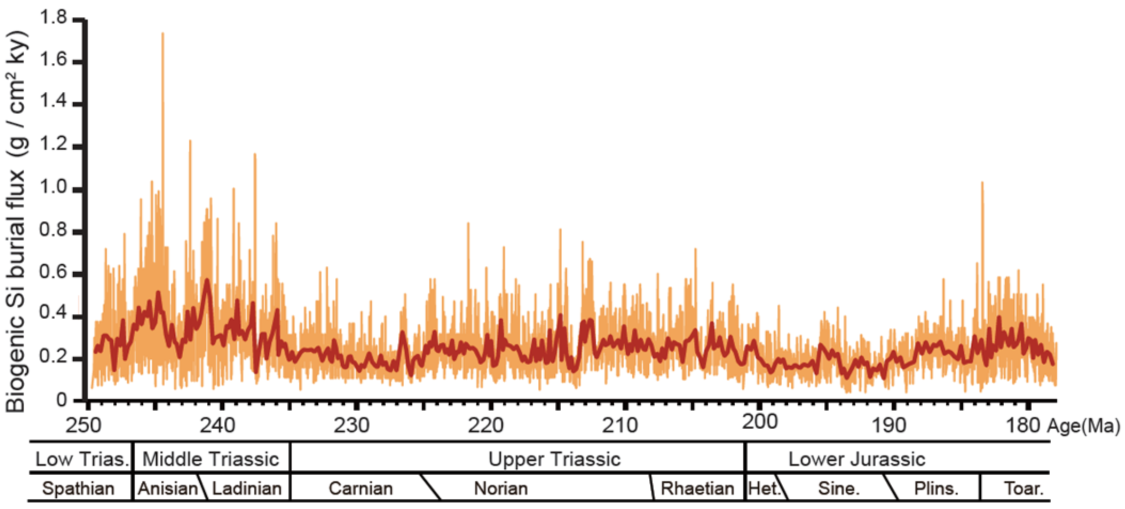

Supplementary Figure 3. Temporal variations in the biogenic silica (BSi) flux during each precession cycle (thin line) and 405 kyr cycles (bold line) of the early Mesozoic bedded chert sequence in Inuyama area, central Japan. Astrochronologic age model is from Ikeda and Tada (2014).

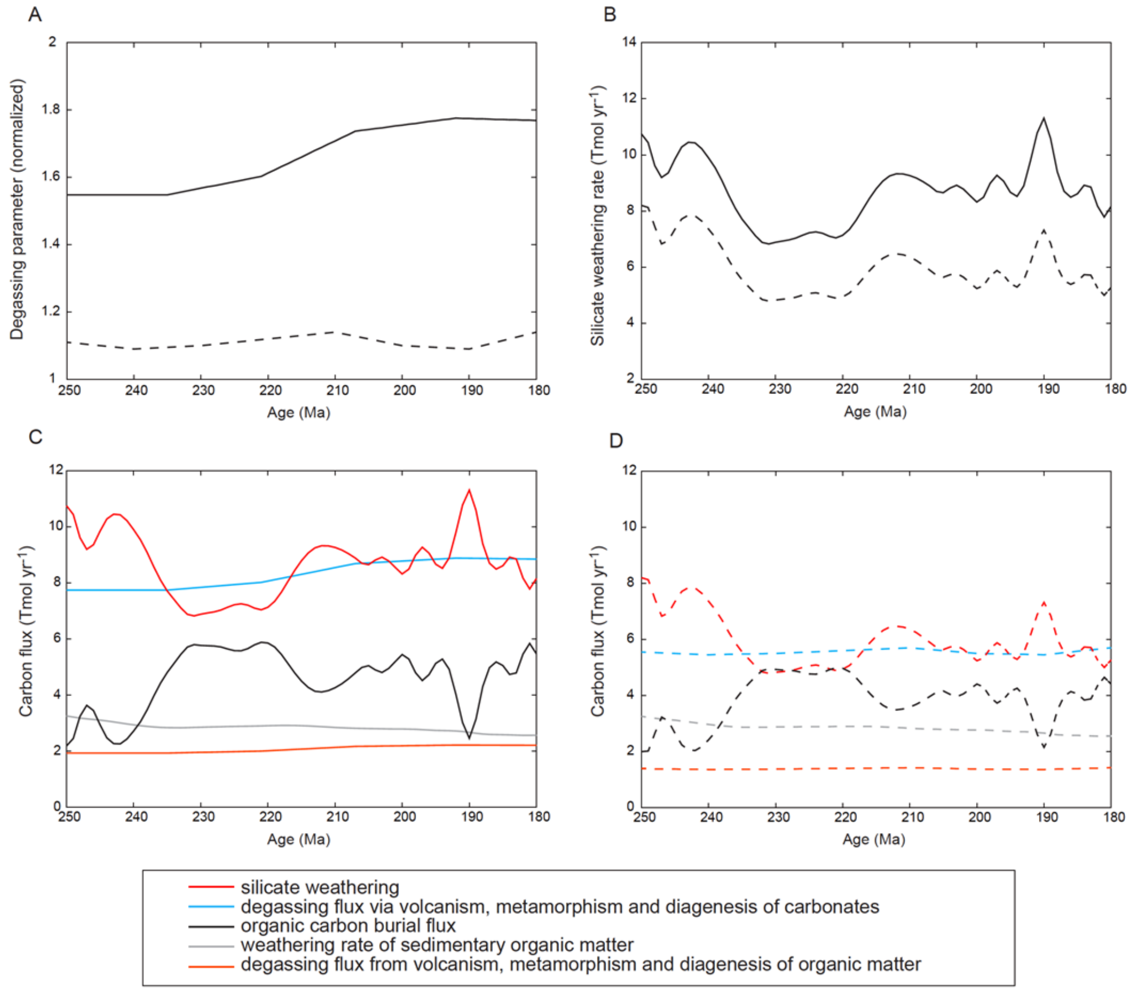

Supplementary Figure 4. GEOCARBSULFvolc model output for different degassing scenarios. (A) Global CO_2 degassing versus time. Black dashed line shows relative CO_2 degassing rate, used as input for GEOCARBSULFvolc (Berner, 2009; Royer et al., 2014) (standard run of this study). Solid line shows Van Der Meer et al. (2014)'s degassing parameter taking into account total subduction zone length (we assumed a constant rate before 235 Ma in the absence of data). (B) Terrestrial silicate weathering rate calculated with an original (solid line) and Van Der Meer et al. (2014)'s degassing parameter (dashed line). (C) Biogeochemical

fluxes of carbon with Van Der Meer et al.'s degassing parameter; red: silicate weathering; blue: degassing flux via volcanism, metamorphism and diagenesis of carbonates; black: organic carbon burial flux; gray: weathering rate of sedimentary organic matter; orange; degassing flux from volcanism, metamorphism and diagenesis of organic matter. (D) Biogeochemical fluxes of carbon given an original degassing parameter.

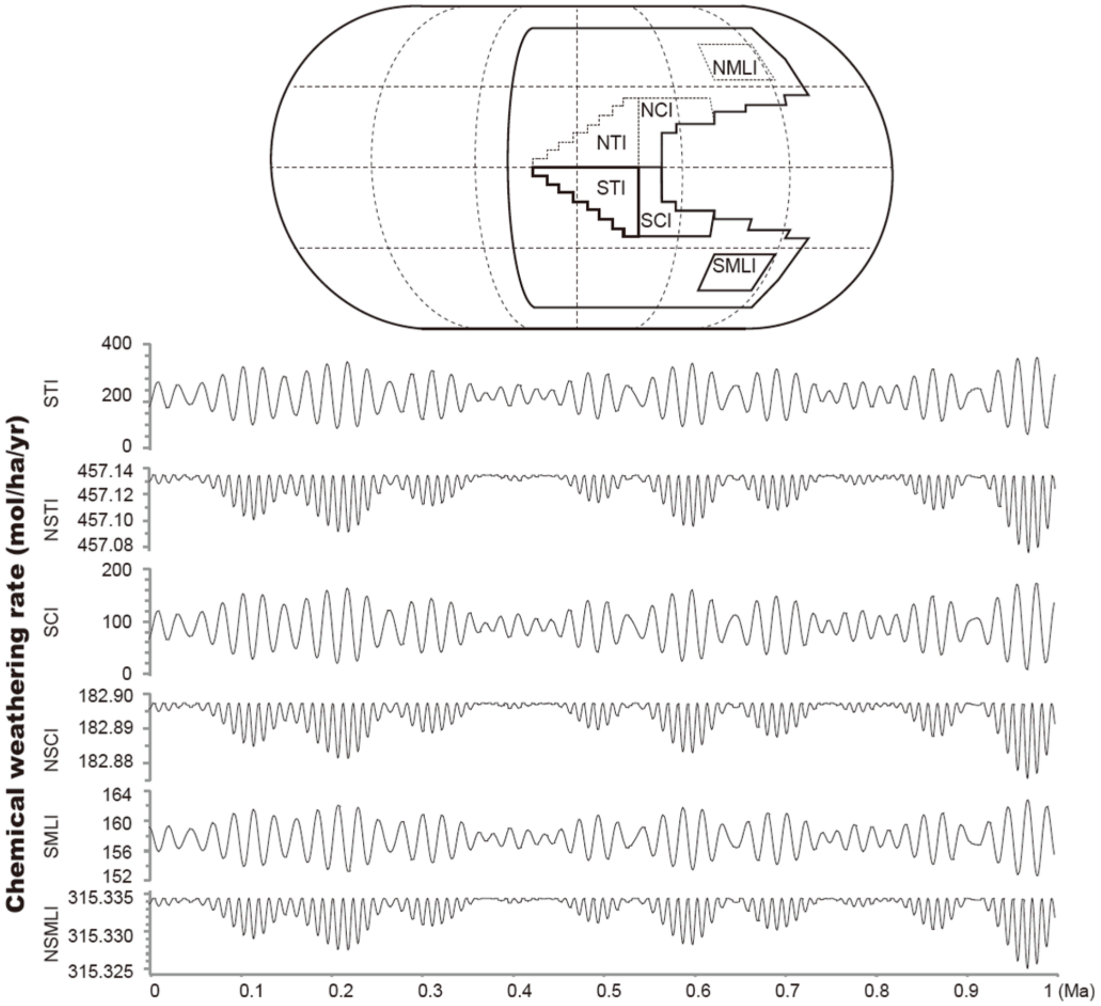

Supplementary Figure 5. **Temporal variations in the model-simulated chemical weathering rate.** The regions in the idealized supercontinent Pangea are the southern tropical interior (STI), the northern and southern tropical interior (NSTI), the southern coast (SC), the northern and southern coast (NSC), the southern middle-latitude interior (SMLI), and the northern and southern middle-latitude interior (NSMLI) (Kutzbach et al., 1994).

	100 kyr	405 kyr	2000 kyr
STI	0.26	0.089	0.021
NSTI	0.0012	0.00049	0.00016
SC	0.18	0.53	0.022
NSC	0.0033	0.0013	0.0044
SMLI	0.0095	0.0032	0.00063
NSMLI	0.00079	0.00032	0.00010

Supplementary Table 1. Relative amplitudes (%) of model-simulated chemical weathering rate for eccentricity cycles. The regions are the southern tropical interior (STI), the northern and southern tropical interior (NSTI), the southern coast (SC), the northern and southern coast (NSC), the southern middle-latitude interior (SMLI), and the northern and southern middle-latitude interior (NSMLI).

Supplementary References

1. Nesbitt H, Young G. Early Proterozoic climates and plate motions inferred from major element chemistry of lutites. *Nature* **299**, 715-717 (1982).

2. Sugitani K, Mimura K. Redox change in sedimentary environments of Triassic bedded cherts, central Japan: possible reflection of sea-level change. *Geol. Magazine* **135**, 735-753 (1998).

3. Hori SR, Cho C, Umeda H. Origin of cyclicity in Triassic-Jurassic radiolarian bedded cherts of the Mino accretionary complex from Japan. *The Island Arc* **3**, 10 (1993).

4. Algeo TJ*, et al.* Spatial variation in sediment fluxes, redox conditions, and productivity in the Permian–Triassic Panthalassic Ocean. *Palaeogeogr. Palaeoclimatol. Palaeoecol.* **308**, 65-83 (2011).

5. Algeo TJ*, et al.* Changes in productivity and redox conditions in the Panthalassic Ocean during the latest Permian. *Geology* **38**, 187-190 (2010).

6. Leinen M, *et al.* Mineralogy of aeolian dust reaching the North Pacific Ocean: 1. Sampling and analysis. *J. Geophys. Res.* **99**, 21017-21023 (1994).

7. Lawrence CR, Neff JC. The contemporary physical and chemical flux of aeolian dust: A synthesis of direct measurements of dust deposition. *Chem. Geol.* **267**, 46-63 (2009).

8. Oda H, Suzuki H. Paleomagnetism of Triassic and Jurassic red bedded chert of the Inuyama area, central Japan. *J. geophys. res.* **105**, 25 (2000).

9. Ando A, *et al.* Low-latitude and Southern Hemisphere origin of Anisian (Triassic) bedded chert in the Inuyama area. *J. Geophys. Res.* **106**, 1973-1986 (2001).

10. Uno K, *et al.* Paleomagnetism of Triassic bedded chert from Japan for determining the age of an impact ejecta layer deposited on peri-equatorial latitudes of the paleo-Pacific Ocean: A preliminary analysis. *Physics Earth Planet. Interiors* **249**, 59-67 (2015).

11. Uno K, *et al.* Palaeomagnetism of Middle Triassic red bedded cherts from southwest Japan: equatorial palaeolatitude of primary magnetization and widespread secondary magnetization. *Geophys. J. Int.* **189**, 1383-1398 (2012).

12. Ikeda M. Cyclostratigraphy of the Triassic to Jurassic deep-sea sequences in Japan: frequency modulation for stratigraphic correlation. *Tanner, LH, Spiel-mann, JA, Lucas, SG (Eds.), The Triassic System. In: Bull.-NM Mus. Nat. Hist. Sci.* **61**, 259-267 (2013).

13. Hori R, Sakakibara M. A chert-clastic sequence spanning the late Triassic-early Cretaceous period of the Kamuikotan Complex in the Shizunai area, south-central Hokkaido, Japan. *J. Geol. Soc. Japan* **100**, 575-583 (1994).

14. Tamaki M, *et al.* A large latitudinal displacement of a part of Cretaceous forearc basin in Hokkaido, Japan: paleomagnetism of the Yezo Supergroup in the Urakawa area. *J. Geol. Soc. Japan* **114**, 207-217 (2008).

15. Müller RD, *et al.* Age, spreading rates, and spreading asymmetry of the world's ocean crust. *Geochem. Geophys. Geosystem.* **9**, (2008).

16. Kodama K*, et al.* Paleomagnetic results from Arrow Rocks in the framework of paleomagnetism in pre-Neogene rocks from New Zealand. *IN: Spoerli, K. B.; Takemura, A.; Hori, R.S. (eds) The oceanic Permian/Triassic boundary sequence at Arrow Rocks (Oruatemanu), Northland, New Zealand. Lower Hutt: GNS Science. GNS Science monograph* **24**, 177-196 (2007).

17. Hagstrum JT, Murchey BL. Deposition of Franciscan Complex cherts along the paleoequator and accretion to the American margin at tropical paleolatitudes. *Geol. Soc. America Bull.* **105**, 766-778 (1993).

18. Hagstrum JT, *et al.* Equatorial origin for Lower Jurassic radiolarian chert in the Franciscan Complex, San Rafael Mountains, southern California. *J. Geophys. Res.* **101**, 613-626 (1996).

19. Scotese C, Langford R. Pangea and the paleogeography of the Permian. In: *The Permian of Northern Pangea* (eds). Springer (1995).

20. Matsuoka A. Jurassic-Early Cretaceous tectonic evolution of the Southern Chichibu terrane, southwest Japan. *Palaeogeogr. Palaeoclimatol. Palaeoecol.* **96**, 71-88 (1992).

21. Soda K, *et al.* Cyclostratigraphic examination of Middle Triassic (Anisian) bedded chert in the Chichibu Belt from Tsukumi area, eastern Kyushu, Japan. *The Geol. Soc. Japan* **121**, 147-152 (2015).

22. Nishi T. Geology and tectonics of the Sambosan Terrane in eastern Kyushu, southwest Japan—stratigraphy, sedimentological features of the depositional setting of the Shakumasan Group. *Jour. Geol. Soc. Japan* **100**, 199-215 (1994).

23. Takemura A, *et al.* Earliest Triassic radiolarians from the ARH and ARF sections on Arrow Rocks, Waipapa Terrane, Northland, New

Zealand. *IN: Spoerli, K. B.; Takemura, A.; Hori, R.S. (eds) The oceanic Permian/Triassic boundary sequence at Arrow Rocks (Oruatemanu), Northland, New Zealand. Lower Hutt: GNS Science. GNS Science monograph* **24**, 97-107 (2007).

24. Sakuma H*, et al.* High-resolution lithostratigraphy and organic carbon isotope stratigraphy of the Lower Triassic pelagic sequence in central Japan. *The Island Arc* **21**, 79-100 (2012).

25. Berner RA, Raiswell R. Burial of organic carbon and pyrite sulfur in sediments over Phanerozoic time: a new theory. *Geochim. Cosm. Acta* **47**, 855-862 (1983).

26. White AF, Blum AE. Effects of climate on chemical_ weathering in watersheds. *Geochim. Cosm. Acta* **59**, 1729-1747 (1995).

27. Dessert C*, et al.* Erosion of Deccan Traps determined by river geochemistry: impact on the global climate and the $^{87}Sr/^{86}Sr$ ratio of seawater. *Earth Planet. Sci. Lett.* **188**, 459-474 (2001).

28. Oliva P, Viers J, Dupré B. Chemical weathering in granitic environments. *Chem. Geol.* **202**, 225-256 (2003).

29. Kutzbach J, Gallimore R. Pangaean climates: megamonsoons of the megacontinent. *J. Geophys. Res.* **94**, 3341-3357 (1989).

30. Kutzbach JE. Idealized Pangean climates: sensitivity to orbital change. *Geol. Soc. America Special Papers* **288**, 41-56 (1994).

31. White AF, Brantley SL. The effect of time on the weathering of silicate minerals: why do weathering rates differ in the laboratory and field? *Chem. Geol.* **202**, 479-506 (2003).

32. Kutzbach JE. Monsoon climate of the early Holocene: climate experiment with the earth's orbital parameters for 9000 years ago. *Science* **214**, 59-61 (1981).

33. Vollmer T, *et al.* Orbital control on Upper Triassic Playa cycles of the Steinmergel-Keuper (Norian): A new concept for ancient playa cycles. *Palaeogeogr. Palaeoclimatol. Palaeoecol.* **267**, 1-16 (2008).

34. Armendáriz M, *et al.* High-resolution chemostratigraphic records from Lower Pliensbachian belemnites: Palaeoclimatic perturbations, organic facies and water mass exchange (Asturian basin, northern Spain). *Palaeogeogr. Palaeoclimatol. Palaeoecol.* **333**, 178-191 (2012).

35. Bailey T, *et al.* Paleoceanographic changes of the Late Pliensbachian–Early Toarcian interval: a possible link to the genesis of an Oceanic Anoxic Event. *Earth Planet. Sci. Lett.* **212**, 307-320 (2003).

36. Berner RA. Phanerozoic atmospheric oxygen: New results using the GEOCARBSULF model. *American J. Sci.* **309**, 603-606 (2009).

37. Brigaud B*, et al.* Facies and climate/environmental changes recorded on a carbonate ramp: a sedimentological and geochemical approach on Middle Jurassic carbonates (Paris Basin, France). *Sedi. Geol.* **222**, 181-206 (2009).

38. Compston W. The carbon isotopic compositions of certain marine invertebrates and coals from the Australian Permian. *Geochim. Cosm. Acta* **18**, 1IN19-18IN222 (1960).

39. Dera G*, et al.* Climatic ups and downs in a disturbed Jurassic world. *Geology* **39**, 215-218 (2011).

40. Dera G*, et al.* Water mass exchange and variations in seawater

temperature in the NW Tethys during the Early Jurassic: evidence from neodymium and oxygen isotopes of fish teeth and belemnites. *Earth and Planetary Science Letters* **286**, 198-207 (2009).

41. Galfetti T*, et al.* Timing of the Early Triassic carbon cycle perturbations inferred from new U–Pb ages and ammonoid biochronozones. *Earth Planet. Sci. Lett.* **258**, 593-604 (2007).

42. Gill BC, Lyons TW, Jenkyns HC. A global perturbation to the sulfur cycle during the Toarcian Oceanic Anoxic Event. *Earth Planet. Sci. Lett.* **312**, 484-496 (2011).

43. Gómez JJ, Canales ML, Ureta S, Goy A. Palaeoclimatic and biotic changes during the Aalenian (Middle Jurassic) at the southern Laurasian Seaway (Basque–Cantabrian Basin, northern Spain). *Palaeogeogr., Palaeoclimatol., Palaeoecol.* **275**, 14-27 (2009).

44. Gómez J, Goy A, Canales M. Seawater temperature and carbon isotope variations in belemnites linked to mass extinction during the Toarcian (Early Jurassic) in Central and Northern Spain. Comparison with other European sections. *Palaeogeogr. Palaeoclimatol. Palaeoecol.* **258**, 28-58 (2008).

45. Grossman EL*, et al.* Glaciation, aridification, and carbon sequestration in the Permo-Carboniferous: the isotopic record from low latitudes. *Palaeogeogr., Palaeoclimatol., Palaeoecol* **268**, 222-233 (2008).

46. Harazim D*, et al.* Spatial variability of watermass conditions within the European Epicontinental Seaway during the Early Jurassic (Pliensbachian–Toarcian). *Sedimentology* **60**, 359-390 (2013).

47. Hesselbo SP, et al.. A potential global stratotype for the Sinemurian–

Pliensbachian boundary (Lower Jurassic), Robin Hood's Bay, UK: ammonite faunas and isotope stratigraphy. *Geol. Magazine* **137**, 601-607 (2000).

48. Jenkyns HC, et al. Chemostratigraphy of the Jurassic System: applications, limitations and implications for palaeoceanography. *J. Geol. Soc.* **159**, 351-378 (2002).

49. Katz ME, et al. Biological overprint of the geological carbon cycle. *Marine Geology* **217**, 323-338 (2005).

50. Korte C, et al., Palaeoenvironmental significance of carbon- and oxygen-isotope stratigraphy of marine Triassic–Jurassic boundary sections in SW Britain. *Journal of the Geological Society* **166**, 431-445 (2009).

51. Korte C, Hesselbo SP. Shallow marine carbon and oxygen isotope and elemental records indicate icehouse-greenhouse cycles during the Early Jurassic. *Paleoceanography* **26** (2011).

52. Korte C, et al., $\delta^{18}O$ and $\delta^{13}C$ of Permian brachiopods: a record of seawater evolution and continental glaciation. *Palaeogeogr., Palaeoclimatol., Palaeoecol.* **224**, 333-351 (2005).

53. Korte C, Kozur HW, Veizer J. $\delta^{13}C$ and $\delta^{18}O$ values of Triassic brachiopods and carbonate rocks as proxies for coeval seawater and palaeotemperature. *Palaeogeogr. Palaeoclimatol. Palaeoecol.* **226**, 287-306 (2005).

54. Li Q, McArthur J, Atkinson T. Lower Jurassic belemnites as indicators of palaeo-temperature. *Palaeogeogr. Palaeoclimatol. Palaeoecol.* **315**, 38-45 (2012).

55. Longinelli A, Iacumin P, Ramigni M. $\delta\ ^{18}O$ of carbonate, quartz and phosphate from belemnite guards: implications for the isotopic record of old fossils and the isotopic composition of ancient seawater. *Earth Planet. Sci. Lett.* **203**, 445-459 (2002).

56. Martinez M, Dera G. Orbital pacing of carbon fluxes by a □9-My eccentricity cycle during the Mesozoic. *Proc. Nat. Acad. Sci.* **112**, 12604-12609 (2015).

57. McArthur J, Howarth R, Shields G. Strontium isotope stratigraphy. *The geologic time scale* **1**, 127-144 (2012).

58. Metodiev L, Koleva-Rekalova E. Stable isotope records ($\delta^{18}O$ and $\delta^{13}C$) of Lower-Middle Jurassic belemnites from the Western Balkan mountains (Bulgaria): palaeoenvironmental application. *Applied geochemistry* **23**, 2845-2856 (2008).

59. Meyer KM, Yu M, Lehrmann D, Van de Schootbrugge B, Payne J. Constraints on Early Triassic carbon cycle dynamics from paired organic and inorganic carbon isotope records. *Earth Planet. Sci. Lett.* **361**, 429-435 (2013).

60. Muttoni G, et al. Middle–Late Triassic (Ladinian–Rhaetian) carbon and oxygen isotope record from the Tethyan Ocean. *Palaeogeogr. Palaeoclimatol. Palaeoecol.* **399**, 246-259 (2014).

61. Nori L, Lathuilière B. Form and environment of Gryphaea arcuata. *Lethaia* **36**, 83-96 (2003).

62. Nunn EV, et al. Isotopic signals from Callovian–Kimmeridgian (Middle–Upper Jurassic) belemnites and bulk organic carbon, Staffin Bay, Isle of Skye, Scotland. *Journal of the Geological Society* **166**, 633-641 (2009).

63. Podlaha OG, Mutterlose J, Veizer J. Preservation of $\delta^{18}O$ and $\delta^{13}C$ in belemnite rostra from the Jurassic/Early Cretaceous successions. *American J. Sci.* **298**, 324-347 (1998).

64. Price GD, et al. Isotopic evidence for long term warmth in the Mesozoic. *Scientific rep.* **3**, 1438 (2013).

65. Price GD. Carbon-isotope stratigraphy and temperature change during the Early–Middle Jurassic (Toarcian–Aalenian), Raasay, Scotland, UK. *Palaeogeogr. Palaeoclimatol. Palaeoecol.* **285**, 255-263 (2010).

66. Prokoph A, Shields G, Veizer J. Compilation and time-series analysis of a marine carbonate $\delta^{18}O$, $\delta^{13}C$, $^{87}Sr/^{86}Sr$ and $\delta\,^{34}S$ database through Earth history. *Earth Sci. Rev.* **87**, 113-133 (2008).

67. Rosales I, Quesada S, Robles S. Paleotemperature variations of Early Jurassic seawater recorded in geochemical trends of belemnites from the Basque–Cantabrian basin, northern Spain. *Palaeogeogr., Palaeoclimatol. Palaeoecol.* **203**, 253-275 (2004).

68. Rosales I, *et al.* Primary and diagenetic isotopic signals in fossils and hemipelagic carbonates: the Lower Jurassic of northern Spain. *Sedimentology* **48**, 1149-1169 (2001).

69. Royer DL, *et al.* Error analysis of CO_2 and O_2 estimates from the long-term geochemical model GEOCARBSULF. *American J. Sci.* **314**, 1259-1283 (2014).

70. Suan G, Mattioli E, Pittet B, Mailliot S, Lécuyer C. Evidence for major environmental perturbation prior to and during the Toarcian (Early Jurassic) oceanic anoxic event from the Lusitanian Basin, Portugal.

Paleoceanography 23 (2008).

71. Teichert B, Luppold F. Glendonites from an Early Jurassic methane seep—Climate or methane indicators? *Palaeogeogr. Palaeoclimatol., Palaeoecol.* **390**, 81-93 (2013).

72. van de Schootbrugge B*, et al.* Early Jurassic climate change and the radiation of organic-walled phytoplankton in the Tethys Ocean. *Paleobiology* 31, 73-97 (2005).

73. Veizer J*, et al.* $^{87}Sr/^{86}Sr$, $\delta^{13}C$ and $\delta^{18}O$ evolution of Phanerozoic seawater. *Chemical geology* **161**, 59-88 (1999).

74. Wu N, *et al.* $\delta^{34}S$ and $\Delta^{33}S$ records of Paleozoic seawater sulfate based on the analysis of carbonate associated sulfate. *Earth Planet. Sci. Lett.* **399**, 44-51 (2014).

75. Zakharov, et al. The Reconstruction of Late Paleozoic and Mesozoic Marine Environments from Isotopic Data. Dalnauka, Vladivostok., 112pp (2001).

76. Matsuda T, Isozaki Y. Well-documented travel history of Mesozoic pelagic chert in Japan: from remote ocean to subduction zone. *Tectonics* **10**, 475-499 (1991).

77. Ikeda M, Tada R. A 70 million year astronomical time scale for the deep-sea bedded chert sequence (Inuyama, Japan): Implications for Triassic–Jurassic geochronology. *Earth and Planet. Sci. Lett.* **399**, 30-43 (2014).

554
